# Exploiting the Potential of Bioactive Molecules Extracted by Ultrasounds from Avocado Peels—Food and Nutraceutical Applications

**DOI:** 10.3390/antiox10091475

**Published:** 2021-09-16

**Authors:** Beatriz Rodríguez-Martínez, Pedro Ferreira-Santos, Beatriz Gullón, José António Teixeira, Cláudia M. Botelho, Remedios Yáñez

**Affiliations:** 1 Department of Chemical Engineering, Faculty of Science, University of Vigo (Campus Ourense), As Lagoas, 32004 Ourense, Spain; beatriz.rodriguez@uvigo.es (B.R.-M.); reme@uvigo.es (R.Y.); 2 Centre of Biological Engineering, Universidade do Minho, 4710-057 Braga, Portugal; pedrosantos@ceb.uminho.pt (P.F.-S.); jateixeira@deb.uminho.pt (J.A.T.); claudiabotelho@deb.uminho.pt (C.M.B.); 3 Biomedical Research Centre (CINBIO), University of Vigo, University Campus As Lagoas-Marcosende, 36310 Vigo, Spain

**Keywords:** agri-food by-products, bioactive compounds, ultrasound assisted extraction, optimization, biological properties

## Abstract

Natural bioactive compounds from food waste have fomented interest in food and pharmaceutical industries for the past decade. In this work, it purposed the recovery of bioactive avocado peel extract using an environmentally friendly technique: the ultrasound assisted extraction. The response surface methodology was applied in order to optimize the conditions of the extraction, ethanol-water mixtures and time. The optimized extracts (ethanol 38.46%, 44.06 min, and 50 °C) were chemically characterized by HPLC-ESI-MS and FTIR. Its antioxidant ability, as well as, its effect on cell metabolic activity of normal (L929) and cancer (Caco-2, A549 and HeLa) cell lines were assessed. Aqueous ethanol extracts presented a high content in bioactive compounds with high antioxidant potential. The most representative class of the phenolic compounds found in the avocado peel extract were phenolic acids, such as hydroxybenzoic and hydroxycinnamic acids. Another important chemical group detected were the flavonoids, such as flavanols, flavanonols, flavones, flavanones and chalcone, phenylethanoids and lignans. In terms of its influence on the metabolic activity of normal and cancer cell lines, the extract does not significantly affect normal cells. On the other hand, it can negatively affect cancer cells, particularly HeLa cells. These results clearly demonstrated that ultrasound is a sustainable extraction technique, resulting in extracts with low toxicity in normal cells and with potential application in food, pharmaceutical or nutraceutical sectors.

## 1. Introduction

Avocado (*Persea americana* Mill.) is a dicotyledonous plant belonging to the Lauraceae family, native from Central America and Mexico. It is one of the most consumed fruits in the world, with a global production of approximately 6.3 million tonnes in 2018 [1]. Currently, it is cultivated in tropical and subtropical places all over the world, being Mexico as the leading producer with one third of the total worldwide production [2]. In Europe, Spain reached a production of about 90,000 tons in 2018, around 95% of the European output [3]. In recent years, the intensification of the global production and increased consumption of avocado are related to its nutritional value and health benefits [4,5].

Among the avocado varieties within the *P. americana* species (Bacon, Fuerte, Hass, Gwen, etc.), Hass is the most industrialized [6]. The avocado industry is important for the guacamole or oils market, for example [7,8]. This industry generates significant amounts of under-utilized by-products, which, if not managed properly, can cause serious environmental problems [9]. These by-products include peels, seeds, or exhausted pulp representing between 21% and 30% of the total weight of the fruit [10].

The use of avocado by-products as composting [11] or natural adsorbent for the removal of dies [12] has already reported. However, due to its chemical composition this material has great economic potential since its use in the food or medical industry is generating great expectations. Even though its composition will be affected by factors such as the cultivar, the growing conditions or the maturation degree, the literature has reported the presence of high contents of carbohydrates (44–84 wt%), and percentages of lipids (2–6 wt%), protein (3–8 wt%), and minerals (2–6 wt%) [13]. Moreover, avocado peels are a remarkable source of biomolecules including phenolic acids, flavonoids as catechins, procyanidins, flavanols, and hydroxycinnamic and hydroxybenzoic acids [14,15]. Recently, it was reported that avocado peel extracts, due to their chemical composition, have numerous biological activities, namely antioxidant, anti-inflammatory, anti-hypertensive and antiproliferative properties [2,15].

The renewable nature, biodegradability, abundance, and low cost associated with their chemical composition makes avocado peels an interesting sustainable raw material. They can be used for the development of integrated biorefinery approaches, based on sequential environmentally friendly processes, which will reinforce the industrial bio-economy promoting well-being and human health [16]. In this context, avocado peels should be reprocessed, in close association with the conception of circular economy and industrial symbiosis, for the production of chemical platforms or functional molecules, with novel industrial applications in the growing market for “natural products”. In addition, it should be taken into account the great interest of food, pharmaceutical, and cosmetic industries in replacing synthetic compounds, normally associated with harmful health effects, by innocuous natural bioactive compounds with beneficial potential in the management (prevention and treatment) of various medical conditions like cancer, cardiovascular diseases, high blood pressure, diabetes, and neurological disorders [9,17,18,19,20].

A key aspect for the isolation of biomolecules from agro-industrial residues is the selection of the most appropriate technology to obtain a high extraction efficiency and maintain the bioactive characteristics of the recovered molecules. Traditionally, these biomolecules were obtained using extraction procedures such as Soxhlet, hydrodistillation, maceration, among others. However, these methods demand a long processing time and large amounts of toxic organic solvents. In the last decade, ultrasound-assisted extraction (UAE) was considered a safe and green technology for the extraction of bioactive compounds from several renewable raw materials, such as olive trees [21], grape pomace [22], pomegranate peels [23], among others [24]. In sonication, the cavitation bubbles on the plant cell walls’ surface causes their disruption, helping the solvent to penetrate within them, thus increasing the mass transfer and ending in better diffusion into the cell material [25,26]. Moreover, the observed structural modifications are beneficial in reducing the severity of the treatment (shorter reaction times and lower temperatures) [27,28]. Compared with other modern extraction techniques, UAE has several advantages like versatility, simplicity and mild temperature operation. These characteristics are attracting attention, as this technique is one of the most feasible and promising technologies to be implemented at an industrial level [29,30]. However, despite this, UAE was scarcely applied for the recovery of bioactives from avocado peels.

Different operational variables, such as temperature, ultrasonication time, solvent type, solvent-sample ratio, power, and frequency affect the efficiency of UAE [31]. Therefore, precise control of these parameters is necessary for optimal extraction. The response surface methodology (RSM) is a widely mathematical tool employed in the optimization of bioactive compounds extraction process from several by-product sources [21,32,33].

Thus, the objective of the present research was to optimize the UAE conditions, time, and solvent content (aqueous EtOH), for the isolation of phenolic compounds from avocado peels. For this purpose, RSM based on a central composite design (CCD) of two-factor at three-levels was used. The total phenolic content (TPC), the total flavonoid content (TFC) and antioxidant activity, and ABTS, DPPH, and FRAP assays were evaluated. The extract recovered under optimal conditions was analyzed by HPLC ESI-MS and FTIR for the identification of functional compounds. Furthermore, in vitro cell metabolic activity of the avocado peels extracts was determined using normal and cancer cell lines (L929, Caco-2, A549, and HeLa).

## 2. Materials and Methods

### 2.1. Raw Material

Avocado peels (AP) from the Hass variety were obtained in a local restaurant in Ourense (Spain). The AP were washed with tap water and subjected to drying at 50 °C for 24 h in a drying oven (JP Selecta Theroven) until at a constant moisture content (<6%). Then, the dried samples were ground to a particle size between 0.3–1 mm using a Polymix PX-MFC 90D. Finally, the ground AP was stored at −20 °C in plastic bags until use.

### 2.2. Reagents and Chemicals

All chemicals and reagents were of analytical grade. Ethanol, methanol, gallic acid, rutin, trolox (6-hydroxy-2,5,7,8-tetramethylchroman-2-carboxylic acid), Folin-Ciocalteu reagent, ABTS (2,2′-azino-di(3-ethylbenzothiazoline-6-suslfonic acid), TPTZ (2,4,6-tri(2-pyridyl)-S-triazine), DPPH (2,2-diphenyl-1-picrylhydrazyl), sodium carbonate, sodium acetate 3-hydrate, potassium persulfate, acetic acid, hydrochloric acid, iron(III) chloride hexahydrate, sodium hydroxide and Dulbecco’s Modified Eagle Medium (DMEM), foetal bovine serum (FBS), penicillin-streptomycin solution, resazurin sodium salt, and dimethyl sulfoxide (DMSO, ≥99.9%) were obtained from Sigma-Aldrich (Barcelona, Spain).

### 2.3. Ultrasound Assisted Extraction (UAE)

The UAE assays were performed in an ultrasonic bath (Branson CPX 3800 H) at a constant temperature of 50 °C and running at a frequency of 37 kHz. This temperature was selected based on preliminary studies (data not shown) and other related research [21,32,33]. For this, AP was placed in a 100 mL beaker with a fixed liquid–solid ratio (LSR) of 8:1 mL/g. When the extraction is finished, the extracts were recovered by vacuum filtration and stored at −20 °C until further analysis.

### 2.4. Experimental Design

A two-factor and three-level central composite design (CCD) with three replicates at the central point (11 experiments) was built to optimize the extraction conditions. Table 1 displays the fixed, independent, and dependent variables selected as well as the nomenclature and their value or range. A summary of the experimental conditions of the set of experiments proposed is also listed in Table 2. Experimental data were fitted applying the regression analysis function of Microsoft Excel’s Data Analysis Add-In, USA. The adequacy of the model was demonstrated by appraising the coefficient of determination (R^2^), the significance of the regression coefficients, and the F-test value obtained from the analysis of variance.

### 2.5. Selection of Optimal UAE Conditions and Validation of the Model

For the purpose of obtaining an extract rich in phytochemicals with high bioactivity (evaluated by DPPH, ABTS, and FRAP), a multi-response surface optimization was applied to determine the extraction conditions which maximize all response variables simultaneously. This optimization was performed using the desirability function of the software Statgraphics Centurion version XVI (Statpoint Technologies Inc., Warrenton, VA, USA). The impact coefficient for all responses was the same. The validity of the model was confirmed experimentally by carrying out three assays under the optimal UAE conditions.

### 2.6. Analyses of the Avocado Peels Extracts (APE)

#### 2.6.1. Determination of Total Phenolic (TPC) and Flavonoid Content (TFC)

Assays for TPC were performed following the procedure of Folin-Ciocalteau and the TFC through the colorimetric method employed by Blasa et al. [34]. Both analyses were carried out in three replicates and the results were conveyed as milligrams of gallic acid equivalent (GAE) per g dry AP for TPC and milligrams of rutin equivalent (RE) per g dry AP for TFC.

#### 2.6.2. Antioxidant Activity

The antioxidant potential of obtained APE was established employing the following methods: DPPH radical scavenging ability assay, ABTS^+^ scavenging activity assay and ferric reducing antioxidant power (FRAP). The assays were performed employing the same protocols described in detail by Gullón et al. [32]. For the three methods, three replicates from each experiment were analyzed and the results were recorded in milligram of Trolox equivalents (TE)/g dried AP.

### 2.7. Characterization of the Selected Extract

In order to have a detailed chemical composition and structural profile of bioactive compounds present in APE obtained under the optimized conditions, different analytical techniques were used including HPLC-ESI-MS and FTIR.

#### 2.7.1. HPLC-ESI-MS

A tentative identification of APE phenolic compounds recovered under optimal extraction conditions was carried out by liquid chromatography coupled with trapped ion mobility spectrometry and TOF high-resolution mass spectrometry (HPLC TOF MS). The sample was injected into a ZORBAX Eclipse XDB-C18 rapid resolution HD (2.1 × 100 mm 1.8 Micron de Agilent) and LC separation were carried out on an Elute HPLC (Bruker Daltonics). The mobile phases employed were: 0.1% aqueous formic acid (solvent A) and 0.1% formic acid in acetonitrile (solvent B) at a flow rate of 0.4 mL/min. The linear gradient was: 2% solvent B over 2 min, from 2% to 30% solvent B over 13 min, 30% to 100% solvent B over 2 min, 100 % solvent B over 4 min, 100% to 2% solvent B over 1 min and then isocratically 2% solvent B for 2 min. Ions were generated by an ESI source in negative ion mode. The working conditions were the following: 3000 V capillary voltage, 500 V end plate offset, 8.0 L/min dry gas, 2 bar nebulizer pressure, 220 °C dry heater. Identification of metabolites was based on the accurate mass data, isotopic pattern matching (mSigma value), retention time (when the standard was available) and the compounds reported in the literature.

#### 2.7.2. ATR-Fourier Transform Infrared Spectroscopy

An ALPHA II-Bruker compact FTIR spectrometer (Ettlingen, Germany) was used for the determination of the functional groups and bonding arrangement of the dried APE. The spectrum was recorded over the wavelength range from 4000 to 400 cm^−1^, a resolution of 4 cm^−1^ and 64 scans.

### 2.8. Cell Viability

In vitro cell metabolic activity of the APE was evaluated using four cell lines: normal mouse fibroblast (L929, ATCC^®^ CCL-1), human colorectal adenocarcinoma (Caco-2, ATCC^®^ HTB-37), human lung cancer (A549, ATCC^®^ CCL-185) and human cervix epithelioid carcinoma (HeLa, ATCC^®^ CCL-2). Cells were cultured in Dulbecco’s Modified Eagle Medium (DMEM) with 10% fetal bovine serum (FBS) and 1% penicillin/streptomycin (1% non-essential amino acids were added to Caco-2 culture medium), at 37 °C in a 5% CO_2_ humidified atmosphere. Upon confluence, cells were trypsinized and seeded in a 96-well plate at a density of 1 × 10^5^ cells per well. Different concentrations of the APE (8, 16, 32, 63, 125, 250, 500 and 1000 µg/mL) were prepared in supplemented culture medium and the viability activity was assessed using the resazurin reduction assay [35,36]. Cell viability was carried out using the procedure described in detail by Ferreira-Santos et al. [35]. The % cell viability was computed based on the values of the cell-free medium (blank) and the negative controls (0.5% DMSO). Only a viable cell has the capability to convert the resazurin into the fluorescent resorufin.

## 3. Results and Discussion

### 3.1. Experimental Design Proposed by Studying the Recovery of Bioactive Molecules

In this study, with the purpose of recovering liquid fractions enriched in bioactive compounds from AP, the application of ultrasonic technology in ethanol-water media was selected. Ultrasonic assisted technology has been previously employed in obtaining valuable biomolecules from agri-food by-products [24]. This technique helps to reduce time, solvent and energy spend and can, therefore, contribute to the development of environmentally friendly applications [9]. Moreover, the use of ethanol as the solvent is another advantage, it is cheap, non-toxicity [37], and generally recognized as safe (GRAS) [9].

Preliminary experiments were carried out to identify the most influential variables and their range of action. These assays were used to determine the values for the fixed variables (data not show). Afterwards, an experimental design of 11 experiments was performed, where the chosen independent variables were: ethanol percentage (EtOH, % *v*/*v* or *x*_1_) and time (*t*, min or *x*_2_). The variable ranges considered were 0–80% and 10–60 min, respectively (see Table 1).

So, with the aim to optimize the recovery of the key compounds, TPC, TFC, and antioxidant activities (analyzed by DPPH, ABTS, and FRAP assays) were selected as dependent variables. Table 2 shows the main experimental results obtained for these parameters in the set of experiments proposed in the design. From these results, it should be noted that the ethanol concentration was more influential than time. As a general trend, increasing time had a positive effect in the recovery of phenolic and flavonoid compounds, whereas intermediate concentrations of ethanol allowed reaching high TPC and TFC, both decreasing considerably thereafter. Interestingly, the highest experimental TPC and TFC (with average values of 42.9 mg GAE/g dried AP and 83.9 mg RE/g dried AP, respectively) were obtained in the central point of the design, at intermediate EtOH concentration and reaction times (40% EtOH and 35 min). As can be seen in Table 2, these experimental conditions also resulted in very high experimental antioxidant activities, established by DPPH, ABTS, and FRAP assays, with average values of 74.9, 148.2 and 40.9 mg TE/g dried AP, respectively.

Good correlations between TPC, TFC, and antioxidant activities were previously reported for several substrates using different extraction methods: (i) conventional treatment with ethanol-water of yerba mate [38], coffee silverskin [39], and spent yerba mate [33]; (ii) in acetone extracts of peach fruit [40]; (iii) after ultrasonic ethanol-water treatment of olive tree by-products [21] and grapefruit wastes [41]; (iv) or even in hydrothermal treatment liquors of melon by-products [42].

The effects of the extraction variables depend mostly on the raw material used. For example, a negative effect on variable time on TPC was reported by Segovia et al. [43] using UAE with avocado seeds at different temperatures and powers. On the other hand, Ilaiyaraja et al. [44] and Dahmoune et al. [45] showed a great influence of the percentage of ethanol during the extraction of phytochemicals from *Feronia limonia* fruit, and *Citrus limon* residues, reaching higher yields at medium EtOH contents. García-Castelló et al. [41] have reported the same trend in the recovery of flavonoids from grapefruit wastes by UAE and 0.4 g/g of ethanol-water. However, the influence of this parameter on TPC and TFC was not evaluated in previous studies performed with avocado by-products [2,6,9]. In this context, in the study by Trujillo-Mayol et al. [9] optimization of UAE was proposed for polyphenols recovery, but only the effect of time (15–60 min) and temperature (40–60 °C) was evaluated. On the other hand, our work adds another important variable into the process (ethanol concentration) that has a great impact on the recovery process of phenolic compounds.

### 3.2. Response Surface Methodology Assessment for the Optimization of Bioactive Compounds Recovery from Avocado

For an easier elucidation of the results obtained, response surface methodology (RSM) was employed. For that purpose, dependent variables (listed in Table 3) were correlated with independent variables (EtOH and extraction time) by empirical models, as follows:

(1) $$y_{j}=b_{0j}+\sum_{i=1}^{2}b_{ij}x_{i}+\sum_{i=1}^{2}\sum_{k=1}^{2}b_{ikj}x_{i}x_{k}$$

where *y_j_* (*j* = 1 to 5) reflects the dependent variables; *x_i_* or *x_k_* (*i* or *k*: 1 to 2, *k* ≥ *i*) the normalized, independent variables (defined in Table 1), and *b*_0*j*_…*b_i_k_j_* represent the regression coefficients calculated by multiple regression using the least-squares method from experimental results. The regression coefficients, statistical significance (based on the Students *t*-test) and the statistical significance of model (Fischer’s F parameter) were collected in Table 3. The high R^2^ values (>0.985) and the values of the other statistical parameters stated a close correlation between the experimental and the predicted data, as well as a good significance of the model.

#### 3.2.1. Effect of the Ultrasonic Pre-Treatment on the Total Phenolic Content (TPC)

**Figure 1:**
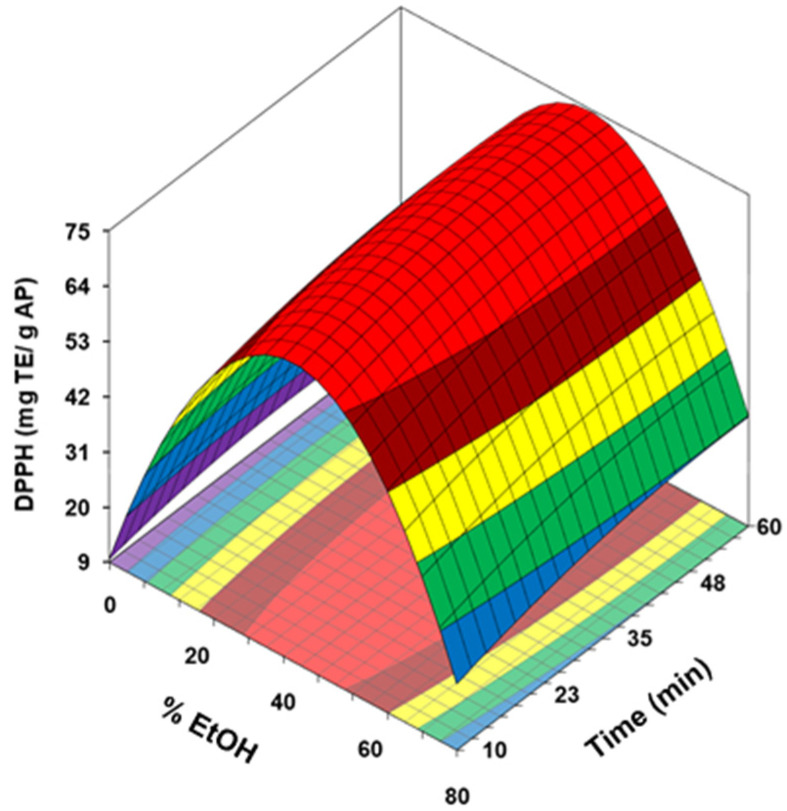
Response surface of total phenolic content (TPC) for AP as a function of ethanol concentration and ultrasonication time.

In agreement with the absolute value of the coefficients shown in Table 3, the quadratic term of the EtOH concentration was the most influential variable on TPC (denoted *y*_1_), followed by the quadratic and lineal terms of the time. The quadratic terms exercised a negative effect on the isolation of the phenolic compounds, whereas the linear effect of the extraction time was positive. Figure 1 shows the relation between the EtOH concentration and extraction time on TPC. This response variable increased with the extraction time up to times close to 42 min; afterward, the model predicted a slight decrease of the TPC. However, EtOH concentrations higher than 40% had a negative impact on the content of phenolic compounds. Therefore, the higher TPC predicted by the model (42.7 mg GAE/g dried AP) was reached at 40–45 min and EtOH concentrations about 40%.

Previous studies also reported similar behavior for the surface responses of TPC with EtOH concentration, reaching the optimum phenolic contents at intermediate ethanol concentrations and then decreasing [14,21,44,45]. This fact would be associated with the negative influence of the quadratic term of EtOH on the phenolic recovery, also observed in other studies previously cited [21,41,44]. Moreover, certain authors highlight the important role of the solvent polarity in the solubilization of phenolic compounds, since these compounds present a wide spectrum of solubilization and, therefore, mixtures of ethanol-water may be more efficient than single solvents [32,44]. In this sense, certain authors emphasize the importance of the similarity of the polarity of solvents and solutes [37] and the decreasing of the solvent dielectric constant caused by ethanol, in enhancing phenolic compounds solubilization and diffusion [41].

#### 3.2.2. Effect of the Ultrasonic Treatment on the Total Flavonoid Content (TFC)

**Figure 2:**
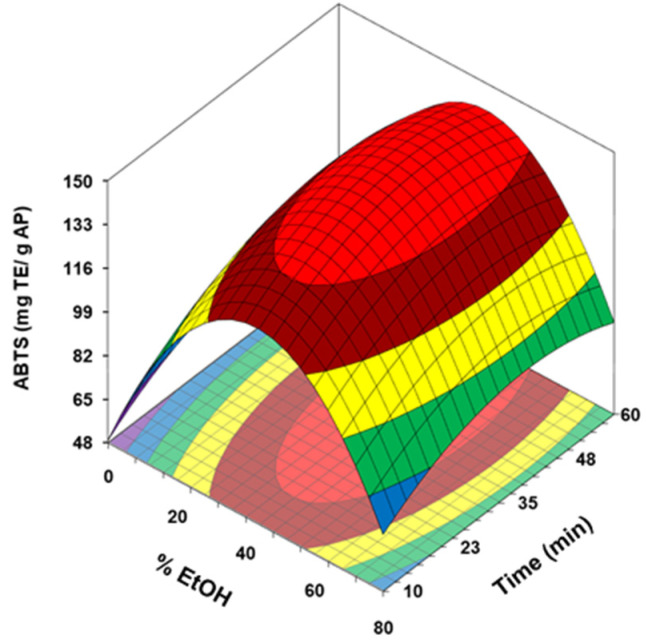
Response surface of total flavonoid content (TFC) for AP as a function of ethanol concentration and ultrasonication time.

Similarly, to the variation pattern observed for the TPC, the quadratic term of the EtOH concentration was the most influential variable on TFC, also following by the quadratic and lineal terms of the time; but in this case, the EtOH concentration had a higher influence. From the regression parameters shown in Table 3, it should be noted that the lineal term of extraction time had a positive effect on the TFC. Conversely, this parameter was negatively affected by the quadratic terms of both factors. Figure 2 depicts the combined influence of the percentage of EtOH and extraction time on the TFC. As can be seen, the calculated TFC ranged from 31.4 to 83.1 mg RE/g dried AP, values predicted in the extraction without ethanol at the lowest time evaluated (10 min) and with 40% ethanol and about 40–42 min, respectively. Therefore, maximum TFC were obtained in similar experimental conditions to the ones previously discussed for the maximum TPC.

Other studies also reported a similar behavior for TFC and a positive effect of ethanol:water mixtures in their recovery. In this context, Martínez-Patiño et al. [21] reported maximum TFC at intermediate ethanol concentrations (closed to 50%). However, a decrease observed at extraction times higher than 12 min, evidence a possible degradation of thermo-sensitive compounds. The same trend of flavonoids extraction with different ethanol concentrations was also described by Xu et al. [37] in *Limonium sinuatum* extracts or by García-Castelló et al. [41] with grapefruit solid residues.

#### 3.2.3. Effect of the Ultrasonic Treatment on Antioxidant Capacity

To assess the impact of the UAE on the antioxidant potential of the extracts obtained from AP, data from DPPH, ABTS, and FRAP assays were included as dependent variables and modelled. As can be deduced for the regression coefficients showed in Table 3, the quadratic term of the EtOH concentration was the most influence variable and had a negative impact in the three response variables. Moreover, the antioxidant capacity measured by ABTS was also negatively influenced by the quadratic term of the extraction time. However, a positive effect of the linear terms of the EtOH concentration and time was obtained in the DPPH and ABTS regression analysis, being higher in both cases for EtOH. On the contrary, this last dependence variable had no influence on the polynomial model obtained for FRAP.

**Figure 3:**
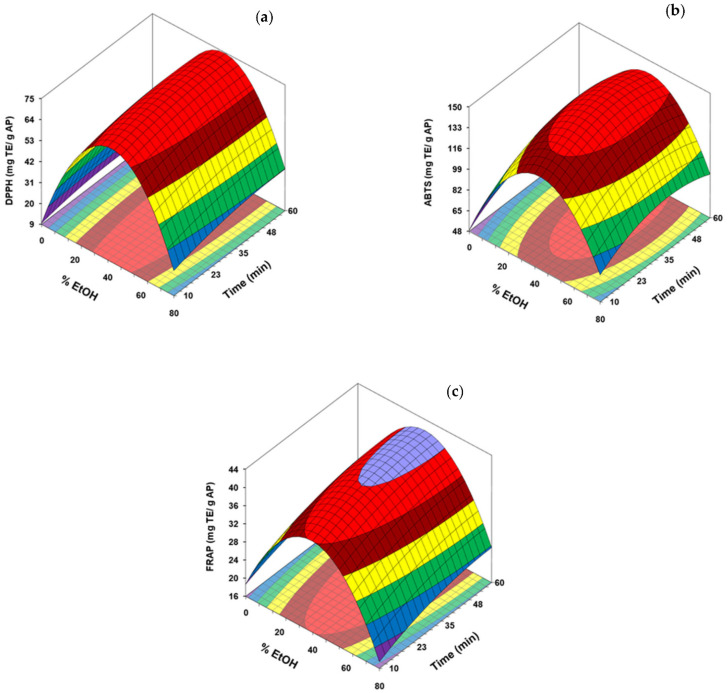
Response surface of DPPH (**a**) ABTS (**b**) and FRAP (**c**) for avocado peel (AP) as a function of ethanol concentration and ultrasonication time.

The response surfaces obtained for the antioxidant potential determined by DPPH, ABTS and FRAP tests are collected in Figure 3 (a, b and c respectively). The values predicted by the model for these parameters ranged from 9.6 and 74.9 for DPPH, between 48.8 and 146.5 for ABTS and 17.5 to 41.6 for FRAP, all expressed in mg TE/g dried AP. These maximum antioxidant activities were obtained at intermediate ethanol concentration (40–44%) and prolonged reaction times, about 50, 45, and 57 min for FRAP, DPPH and ABTS modelling, respectively. As with TPC and TFC, ethanol concentrations higher than 40–44% had a marked negative impact on antioxidant capacity, which would indicate a good correlation between these parameters. The higher values for ABTS were obtained at extraction times close to predicted for maximum TPC and TFC (40–44 min), being necessary to prolong the duration of the treatment up to 50–57 min to reach the highest values of DPPH and FRAP.

García-Castelló et al. [41] also described the same trend of DPPH with ethanol content in grapefruit solid wastes extracts. Xu et al. [37], in a previous study dealing with the UAE of the flower of *Limonium sinuatum*, displayed that the linear and quadratic coefficients of ethanol content were significant on ABTS modelling, whereas the influence of the ultrasound time was not significant. In this case, the negative effect of the quadratic term on the percentage of ethanol was also reported. Araújo et al. [46] also noted a significant effect of the quadratic term of the ethanol content on the isolation of antioxidant compounds in MAE of avocado seeds. In this study, the highest content of phenolic compounds and antioxidant activities were also identified at intermediate percentages of ethanol (close to 50%).

Establishing comparisons between the antioxidant activities collected in literature for avocado by-products is not easy, due to the large number of analytical methods available for their determination and the different extraction conditions.

### 3.3. Process Optimization and Validation of the Model

In this work, for the purpose of achieving an extract with high content of phytochemicals and with high bioactivity, a multiple response optimization applying the desirability function was used to determine the UAE conditions that enabled simultaneous maximization of the five studied responses. The selected conditions resulting in an extract with these characteristics were: ethanol concentration of 38.46% (*v*/*v*) and 44.06 min. Using microwave-assisted extraction, Araújo et al. [19] estimated an optimal ethanol concentration similar to that found here to obtain bioactive compounds from this same residue. In another work, Martínez-Patiño et al. [21] reported a shorter extraction time (15 min) and a higher ethanol percentage (51.3%) as optimal conditions for the ultrasonic treatment of olive mill leaves.

With the purpose to confirm the validity and the adequacy of the model, three trials under optimal conditions were performed. Table 4 collects the predicted and experimental results for all responses analyzed in this study. As can be seen, the experimental data were similar to the predicted response values (the error varied between 3 and 5.6%), which indicates the suitability of the experimental design to optimize the recovery of antioxidants from AP. Under these optimum conditions, the TPC and TFC were of 45.34 mg GAE/g dried AP and 87.56 mg RE/g dried AP, respectively, with DPPH of 73.25 mg TE/g dried AP, ABTS of 160.34 mg TE/g dried AP and FRAP of 44.65 mg TE/g dried AP. Comparing the experimental results, the TPC observed by Tremocoldi et al. [2] after ultrasonic treatment (80% ethanol, 40 KHz, 15 min at 25 °C, LSR of 10 mL/g) of AP of the Hass variety was 63.5 mg GAE/g lyophilized AP. Trujillo-Mayol et al. [9] using the same conditions but increasing the temperature at 60 °C and the LSR at 25 mL/g found a TPC value of around 2.9 times higher than that obtained in our research. Regarding antioxidant activity, in the cited studies, Trujillo-Mayol et al. [9] reported for UAE extracts, values of DPPH and FRAP of 772.2 and 161.7 μgTE/g dried extract, respectively; whereas Tremocoldi et al.’s [2] values of DPPH and ABTS of 310 and 791.5 μmol TE/g of AP lyophilized, respectively, and FRAP of 1175.1 μmol Fe^2+^ g of AP lyophilized. It is important to take into account that the differences detected by different authors in the antioxidant potential of the extracts recovered from AP is affected by different variables, such as the type of cultivar, ripeness stage, and environmental and nutritional growth conditions [10].

In order to have detailed information of the recovered extracts under optimal conditions (ethanol concentration of 38.46% (*v*/*v*) and 44.06 min), these were analyzed to identify the main phenolic compounds, their structural characteristics, and their cytotoxic potential.

### 3.4. Characterization of the Extract Obtained under the Optimum Conditions

#### 3.4.1. HPLC-TOF-MS Analysis

The identification of compounds present in the APE was based on the following criteria: (i) the measurement of the accurate mass of pseudomolecular ion [M-H]- compared to the theoretical mass (the mass error among the theoretical and the measured mass was <10 ppm), (ii) the mSigma value lower than 50, (iii) retention time (when the standard was available) and (iv) the information reported in previous reports [17,19,47]. Table 5 collects the main identified compounds in AP extracts. HPLC-TOF MS analysis allowed to identify different families of compounds in the extracts including phenolic acids, organic acids, and lignans that are associated with the in vitro antioxidant activity previously determined in the APE.

The main phenolic acids identified in this work were 4-hydroxybenzoic acid, chlorogenic acid, benzoic acid, and p-coumaric acid. According to the literature, these compounds were previously reported in avocado peels [17,48,49]. For example, Figueroa et al. [14] used HPLC-Q-TOF MS/MS to detect caffeic acid, p-coumaric acid and different isomers of caffeoylquinic acids derivatives in extracts from avocado fruit peel obtained by accelerated water–ethanol extraction. These compounds present bioactive properties that make avocado by-products suitable sources for the formulation of functional ingredients. In this sense, it has been described that chlorogenic acid presents hypoglycemic, hypolipidemic, and antioxidant properties [50] and p-coumaric acid exhibits antimicrobial activity.

Regarding flavonoids, compounds belonging to different subclasses were identified: flavanols (catechin, epigallocatechin 3-coumarate) flavonols (quercetin-dihexose, quercetin *O*-arabinosyl-glucoside, rutin, quercetin, quercetin 3-glucoside, quercetin 3-glucuronide, kaempferol-hexose, kaempferol *O*-glucosyl-rhamnoside), flavones (luteolin 7-*O*-(2″-*O*-pentosyl) hexoside), flavanones (naringenin) and flavanonols (taxifolin). The identification of these compounds in our extract is in agreement with the flavonoids found by other authors in avocado peel extracts [15,17,46,47]. Multiple works have confirmed the beneficial health properties of flavonoids consumption including their role as antioxidant, hypolipidemic and anti-obesity, antimicrobial, neuroprotective, among others [51]. For example, Tremocoldi et al. [2] demonstrated that different flavonoids, such as epicatechin, trans-5-*O*-caffeoyl-d-quinic acid, catechin and epicatechin, exhibited anti-inflammatory and cytotoxic properties. Quercetin improves insulin-stimulated glucose uptake in mature adipocytes, and rutin presents hypoglycemic and hypolipidemic properties [50].

Different procyanidins were also identified in the extracts obtained by ultrasonication of avocado peels. Several reports have shown the presence of these condensed flavonoids in avocado by-products. For instance, Rosero et al. [47] studied the procyanidins in the seeds and peels of ‘Nariño’ avocado, providing the identification of types A (1 dimer and 1 trimer) and 7 of types B (four dimers and three trimers). Kosińska et al. [48] found in the Hass variety two type B procyanidin dimers and another type A. Various studies have reported that procyanidins present interesting bioactivities, such as modulate antioxidant enzymatic activities [52], exhibit chemoprotective properties against cancer [53,54], and prevent urinary tract infections [54], among other bioactivities.

The presence of the lignan nudiposide and neosakuranetin is in consonance with previous research on avocado peel [17]. Other families of compounds such as organic acids (quinic acid), phenolic alcohol derivatives (tyrosol-hexoside-pentoside) were tentatively identified.

#### 3.4.2. ATR-FTIR Spectra Analysis

**Figure 4:**
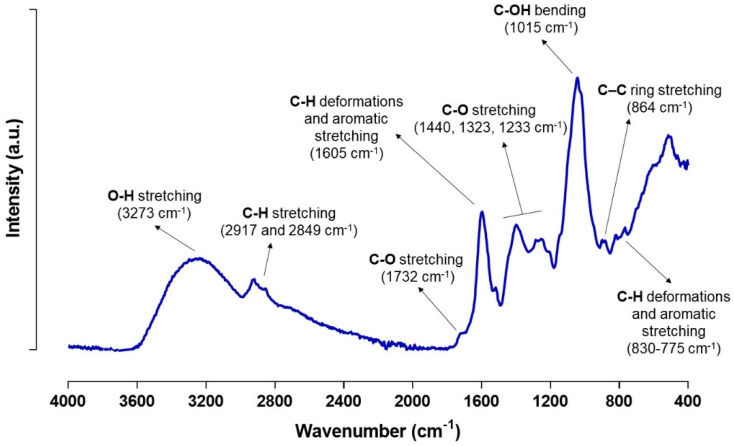
ATR-FTIR spectra of freeze-dried APE.

Figure 4 displays the FTIR spectra of the optimized freeze-dried APE at a wavelength ranging from 400–4000 cm^−1^. The broad peak in the region of 3600–3000 cm^−1^ (3273 cm^−1^) can be accredited to the O–H stretching of phenolic and aliphatic structures. The hydrophobic phenolic compounds signature is represented by the peaks at 2917 and 2849 cm^–1^ [35]. The band at 1735 cm^−1^ is attributed to the carbonyl C–O from the ester group related to esterified pectins. The peaks at 1605 and 1323 cm^−1^ are originated by the vibration of the aromatic skeletal, while the peak at 1232 cm^−1^ is due to the skeletal vibrations of the aromatic ring with C–O stretching. According to the literature the bands at 1440 and 1017 cm^−1^ are associated to polysaccharides, pectins, and sugars [55]. The chemical characterization of the avocado extract demonstrated the presence of phenolic compounds, which were detected by the FTIR spectra due to the stretching and bending vibrations of –CH from aromatic rings assigned to the peaks between 864 to 775 cm^−1^. Similarly, the presence of flavonoids and polyphenols were also observed due to the presence of vibrational bands assigned to the bonds O–H, C–H, C=C ring, C–OH, and C–C [56]. The FTIR spectra results are supported by the data previously described, as well as by the literature which refers the presence of pectins, starch, sugars, lignin, and phenolic compounds in avocado by-products resulting extracts [5,15].

#### 3.4.3. Cell Viability

**Figure 5:**
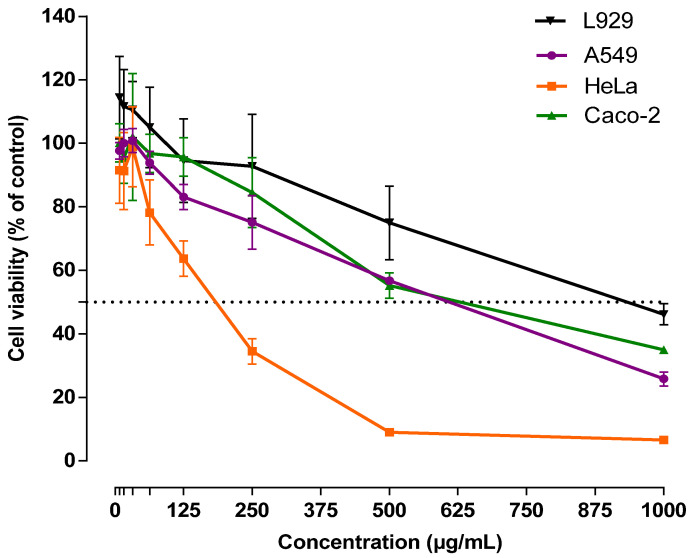
Cellular viability (%) of APE against normal mouse fibroblast (L929), human colorectal adenocarcinoma (Caco-2), human lung cancer (A549), and human cervix epithelioid carcinoma (HeLa).

Some natural molecules from plants and by-products can be considered harmful to consumers’ health. So, cytotoxic studies are necessary to evaluate the potential toxic effect of plant extracts [35,57]. In this study, the effect of APE was evaluated on normal mouse fibroblast cells (L929). As can be seen in Figure 5, the data shows that APE induces a low cytotoxic effect in normal fibroblasts, reporting IC_50_ values of 908.6 µg/mL.

Similar results were described by other authors, which showed that avocado ethanolic extract by-products did not have a toxic effect on mouse macrophage RAW 264.7 cells. This result corroborates those obtained in this study, in which even at higher doses, APE show low toxicity. These results indicate that these extracts are safe for human consumption [2].

To this day, cancer remains one of the leading causes of death worldwide. In this sense, the quest for safer strategies to be applied either as mono or adjunctive therapy with traditional narcotics is becoming a priority in anticancer research [58]. Natural products or their derivatives are promising alternatives for anti-cancer therapeutics. Some studies have confirmed that polyphenol-rich extracts from natural resources possess health benefits by delaying tumor onset [59]. The anticarcinogenic properties of these molecules are related with their capacity to inhibit cancer cell proliferation, angiogenesis, metastasis, apoptosis, and inflammation [60]. In vitro cytotoxic characteristics of AP extracts against different tumor cell lines such as breast, colon, liver, lungs, leukemia, oral, ovary, and prostate, were largely studied by other authors [58,61,62,63,64,65,66]. In the present work, the metabolic activity was quantified by the conversion of resazurin to resorufin upon the contact with various concentrations of APE (0 to 1000 µg/mL) for 24 h. It is important to mention that the resazurin test is based on the ability of living cells to transform resazurin to resorufin [67]. This ability is correlated with cell viability and cell number/proliferation. The basis for this understanding is that the higher the conversion of the dye, the higher is the cell number. So, taking into consideration this fact, it is possible to interpret the results observed as the extracts’ influence on cellular proliferation.

The four cell lines showed a dose-response dependent effect (Figure 5). Nonetheless, the behavior of non-cancer L929 cells was different from that of tumour cells (A549, HeLa and Caco-2). Extracts at low concentration (8 to 73 µg/mL) favored L929 metabolic activity, while, for the same concentration, the tumour cells exhibited a reduction on their metabolic activity, which may be an indication of low proliferation.

The most important finding was the ability of the extract to act differently on cancer cells, as these were negatively influenced and the non-tumour cells were not. These facts are confirmed by the lower IC50 values for tumour cells (HeLa 169.2 µg/mL; A549 635.9 µg/mL; Caco-2 670.1 µg/mL) while the non-tumour cells present a higher IC50 (L929 908.6 µg/mL). As it is possible to see, these extracts show to be highly effective against cells of cervix epitheloid carcinoma (HeLa). Moreover, based on the results collected, it is hypothesized that the selectivity of APE is attributed to a synergistic effect of several molecules.

When analyzing the extracts’ composition, it was possible to observe that they are rich in antioxidant phenolic compounds such as catechins, quercetins, kaempferol, phenolic acids, etc. (see Table 5). Most of these compounds have a negative effect in various types of cells. For example, the findings of a study conducted by Sun et al. [68] stated that catechins can adequately inhibit growth of A549 cells via regulating its cell cycle arrest or indirectly through the p21 signaling pathway.

In another study, epigallocatechin gallate inhibited the proliferation and activation of the epidermal growth factor receptor and human epidermal growth factor receptor-2 signalling pathways in human colon cancer cells (Caco-2, HCT116, HT29, SW480 and SW837) [69].

Some studies have indicated that extracts rich in kaempferol are associated with a decreased risk of developing some types of cancer, in particular, skin, liver, and colon. Several mechanisms of action were described for this phenolic compound including apoptosis, cell-cycle arrest at the G2/M phase, downregulation of epithelial-mesenchymal transition (EMT)-related markers, and phosphoinositide 3-kinase/protein kinase B signaling pathways [69].

Quercetin, is a distinctive antioxidant flavonoid that has a well-documented role in management of different human cancers. Quercetin exhibits direct apoptotic effects on tumour cells and thus can prevent the development of many human cancers. The anticancer properties of quercetin were described in many in vitro and in vivo studies that involved some cell lines and animal models. Moreover, the highly toxic effect of quercetin against cancer cells is accompanied with little or no side effects or harm to normal cells [70]. Overall, these results open the path to the potential use of APE in the medical or nutraceutical sectors.

## 4. Conclusions

The work clearly demonstrates that UAE is a good technique for the extraction of biomolecules from by-products of the agroindustry. Upon the variables involved in this extraction method, it is possible to obtain extracts with content in phenolic and flavonoid compounds. Moreover, this technique does not result in the loss of bioactivity of the recover compounds. As it was demonstrated the APE presents high antioxidant activity and low cellular toxicity in normal cells, on the other hand this extract can negatively affect cancer cells, particularly HeLa cells.

The results presented in this work confirm that APE is safe for human consumption, and can be used as a food preservative, antioxidant, or as a bioactive/functional ingredient. Furthermore, the selectivity demonstrated by APE regarding normal and cancer cells opens the opportunity for its use in the pharmaceutical area.

## Figures and Tables

**Table 1:** Experimental variables used in this study.

Variable	Definition and Units	Nomenclature	Value or Range
Fixed	Liquid to solid ratio of extraction (*v*/*w*)	LSR	8 mL/g
	Frequency Temperature		37 kHz 50 °C
Independent	Ethanol concentration (% *v*/*v*)	EtOH	0–80%
	Extraction time (min)	tE	10–60 min
Dependent	Total phenolic content (mg GAE/g dried AP)	TPC or *y*_1_	
	Total flavonoid content (mg RE/g dried AP)	TFC or *y*_2_	
	DPPH• radical scavenging activity (mg TE/g dried AP)	DPPH or *y*_3_	
	ABTS cation radical scavenging activity (mg TE/g dried AP)	ABTS or *y*_4_	
	Ferric reducing antioxidant power (mg TE/g dried AP)	FRAP or *y*_5_	

**Table 2:** Operational conditions assayed (expressed in terms of coded and uncoded independent variables) and experimental values obtained for response variables.

Experiment	1	2	3	4	5	6	7	8	9	10	11
Independent Variables											
EtOH (%) or *x*_1_	0(−1)	0(−1)	0(−1)	80(1)	80(1)	80(1)	40(0)	40(0)	40(0)	40(0)	40(0)
E_t_ (min) or *x*_2_	10(−1)	35(0)	60(1)	10(−1)	35(0)	60(1)	10(−1)	60(1)	35(0)	35(0)	35(0)
Dependent Variables											
TPC or *y*_1_ (mg GAE/g dried AP)	18.1	19.9	18.0	15.0	18.9	20.1	36.4	41.8	42.3	42.9	43.6
TFC or *y*_2_ (mg RE/g dried AP)	31.8	37.1	32.6	34.8	37.1	42.2	72.7	79.9	82.2	84.3	85.3
DPPH or *y*_3_ (mg TE/g dried AP)	11.1	13.0	11.9	24.8	26.2	31.0	64.2	74.9	73.4	75.3	76.1
ABTS or *y*_4_ (mg TE/g dried AP)	54.2	64.8	67.3	73.8	87.7	83.1	114.7	138.8	146.7	148.1	149.7
FRAP or *y*_5_ (mg TE/g dried AP)	18.7	23.5	22.4	19.3	19.6	24.4	34.2	41.8	40.2	42.8	39.7

**Table 3:** Analysis of the variance (ANOVA) of the fitted second-order polynomial models.

Coefficient	*y*_1_	*y*_2_	*y*_3_	*y*_4_	*y*_5_
*b*_0_	42.45 ^a^	82.71 ^a^	73.77 ^a^	145.07 ^a^	40.37 ^a^
*b*_1_	−0.31	2.09	7.68 ^a^	9.71 ^b^	−0.20
*b*_2_	1.71 ^b^	2.56 ^c^	2.95 ^c^	7.76 ^b^	2.73 ^b^
*b*_12_	1.32 ^c^	1.67	1.36	−0.96	0.37
*b*_11_	−22.36 ^a^	−43.72 ^a^	−52.43 ^a^	−64.15 ^a^	−18.02 ^a^
*b*_22_	−2.64 ^b^	−4.57 ^c^	−2.50	−13.67 ^b^	−1.54
R^2^	0.995	0.992	0.994	0.985	0.978
F-exp	222.6	130.3	182.9	66.9	44.96
Significance level (%)	99.999	99.997	99.998	99.986	99.963

^a^ Significant coefficient at the 99% confidence level. ^b^ Significant coefficient at the 95% confidence level. ^c^ Significant coefficient at the 90% confidence level.

**Table 4:** Validation of the predicted values at optimal conditions of UAE.

	TPC (mg GAE/g Dried AP)	TFC (mg RE/g Dried AP)	DPPH (mg TE/g Dried AP)	ABTS (mg TE/g Dried AP)	FRAP (mg TE/g Dried AP)
Predicted value	43.65	84.87	76.61	150.21	42.26
Experimental value ^a^	45.34 ± 1.7	87.56 ± 1.2	73.25 ± 3.4	160.34 ± 65.7	44.65 ± 2.7

^a^ Mean ± standard deviation (SD) of three determinations (n = 3) from three extract replications.

**Table 5:** Tentative identification of phenolic compounds in AP extracts.

Proposed Compounds	Class/Subclass	Molecular Formula	*m*/*z* Meas.	RT (min)	mSigma
Quinic acid	Organic acid	C_7_H_12_O_6_	191.0560	4.54	10.6
Tyrosol-hexoside-pentoside	Phenylethanoids	C_19_H_28_O_11_	431.1557	4.8	17.7
4-Hydroxybenzoic acid	PA/HB	C_7_H_6_O_3_	137.0241	5.04	10
Chlorogenic acid/caffeoyl-quinic acid	PA/HC	C_16_H_18_O_9_	353.0879	6.62	20.6
Benzoic acid	PA/HB	C_7_H_6_O_2_	121.0293	6.79	1.7
Procyanidin trimer B	FL/flavanol oligomer	C_45_H_38_O_18_	865.1973	6.86	48.2
Procyanidin dimer B	FL/flavanol oligomer	C_30_H_26_O_12_	577.1338	7.01	4.7
Catechin	FL/flavanol	C_15_H_14_O_6_	289.0717	7.99	4.1
Quercetin—dihexose	FL/flavanol	C_27_H_30_O_17_	625.1405	9.44	21.7
Quercetin *O*-arabinosyl-glucoside	FL/flavanol	C_26_H_28_O_16_	595.1300	10.46	7.9
Rutin	FL/flavanol	C_27_H_30_O_16_	609.1457	10.76	9.8
Nudiposide	Lignan	C_27_H_36_O_12_	551.2130	11.34	5.6
Epigallocatechin 3-coumarate	FL/flavanol	C_24_H_20_O_9_	451.1031	11.61	8.6
Taxifolin	FL/flavanonol	C_15_H_12_O_7_	303.0507	11.64	3.9
Quercetin	FL/flavanol	C_15_H_10_O_7_	301.0352	11.82	25.6
Quercetin 3-glucoside	FL/flavanol	C_21_H_20_O_12_	463.0881	11.85	4.3
Quercetin 3-glucuronide	FL/flavanol	C_21_H_18_O_13_	477.0674	11.94	12.7
Procyanidin dimer A	FL/flavanol oligomer	C_30_H_24_O_12_	575.1189	12.45	12
Kaempferol-hexose	FL/flavanol	C_21_H_20_O_11_	447.0931	13.14	22.9
Luteolin 7-*O*-(2″-*O*-pentosyl) hexoside	FL/flavone	C_26_H_28_O_15_	579.1354	13.49	9.1
Procyanidin trimer A	FL/flavanol oligomer	C_45_H_36_O_18_	863.1812	14.03	58.5
Kaempferol *O*-glucosyl-rhamnoside	FL/flavanol	C_27_H_30_O_15_	593.1505	14.49	12.5
Neosakuranetin	FL/chalcone	C_22_H_24_O_10_	44731298	14.62	11.9
p-coumaric acid	PA/HC	C_9_H_8_O_3_	163.0399	16.32	6.5
(±)-Naringenin	FL/flavanone	C_15_H_12_O_5_	271.0611	16.69	14.1

* Abbreviations: PA: phenolic acid; HB: hydroxybenzoic acid; HC: hydroxycinnamic acid; FL: flavonoids.

## Data Availability

Data is contained within the article.

## References

[1] Altendorf S. (2019). Major Tropical Fruits Market Review 2017.

[2] Tremocoldi M.A., Rosalen P.L., Franchin M., Massarioli A.P., Denny C., Daiuto É.R., Paschoal J.A.R., Melo P.S., De Alencar S.M. (2018). Exploration of Avocado By-Products as Natural Sources of Bioactive Compounds. PLoS ONE.

[3] Bedford D., Claro J., Giusti A.M., Karumathy G., Lucarelli L., Mancini D., Marocco E., Milo M., Yang D. (2018). Food Outlook.

[4] Dorantes-Álvarez L., Ortiz-Moreno A., García-Ochoa F. (2012). Avocado. Tropical and Subtropical Fruits: Postharvest Physiology, Processing and Packaging.

[5] Del Castillo-Llamosas A., Del Río P.G., Pérez-Pérez A., Yáñez R., Garrote G., Gullón B. (2021). Recent Advances to Recover Value-Added Compounds from Avocado by-Products Following a Biorefinery Approach. Curr. Opin. Green Sustain. Chem..

[6] Melgar B., Dias M.I., Ciric A., Sokovic M., García-Castelló E.M., Rodríguez-López A.D., Barros L., Ferreira I.C.R.F. (2018). Bioactive Characterization of *Persea Americana* Mill. by-Products: A Rich Source of Inherent Antioxidants. Ind. Crop. Prod..

[7] Palou E., Hernández-Salgado C., López-Malo A., Barbosa-Cánovas G.V., Swanson B.G., Welti-Chanes J. (2000). High Pressure-Processed Guacamole. Innov. Food Sci. Emerg. Technol..

[8] Woolf A.B., Wibisono R., Farr J., Hallett I., Richter L., Oey I., Wohlers M., Zhou J., Fletcher G.C., Requejo-Jackman C. (2013). Effect of High Pressure Processing on Avocado Slices. Innov. Food Sci. Emerg. Technol..

[9] Trujillo-Mayol I., Céspedes-Acuña C., Silva F.L., Alarcón-Enos J. (2019). Improvement of the Polyphenol Extraction from Avocado Peel by Assisted Ultrasound and Microwaves. J. Food Process Eng..

[10] Araújo R.G., Rodríguez-Jasso R.M., Ruiz H.A., Pintado M.M.E., Aguilar C.N. (2018). Avocado By-Products: Nutritional and Functional Properties. Trends Food Sci. Technol..

[11] González-Fernández J.J., Galea Z., Álvarez J.M., Hormaza J.I., López R. (2015). Evaluation of Composition and Performance of Composts Derived from Guacamole Production Residues. J. Environ. Manag..

[12] Palma C., Lloret L., Puen A., Tobar M., Contreras E. (2016). Production of Carbonaceous Material from Avocado Peel for Its Application as Alternative Adsorbent for Dyes Removal. Chin. J. Chem. Eng..

[13] Bressani R., Rodas B., Ruiz A.S. (2009). La Composición Química, Capacidad Antioxidativa y Valor Nutritivo de La Semilla de Variedades de Aguacate. CONCYT-Secretaría Nac. Cienc. Tecnol..

[14] Figueroa J.G., Borrás-Linares I., Lozano-Sánchez J., Quirantes-Piné R., Segura-Carretero A. (2018). Optimization of Drying Process and Pressurized Liquid Extraction for Recovery of Bioactive Compounds from Avocado Peel By-Product. Electrophoresis.

[15] Salazar-López N.J., Domínguez-Avila J.A., Yahia E.M., Belmonte-Herrera B.H., Wall-Medrano A., Montalvo-González E., González-Aguilar G.A. (2020). Avocado Fruit and By-Products as Potential Sources of Bioactive Compounds. Food Res. Int..

[16] Albergamo A., Costa R., Bartolomeo G., Rando R., Vadalà R., Nava V., Gervasi T., Toscano G., Germanò M.P., DʼAngelo V. (2020). Grape Water: Reclaim and Valorization of a by-Product from the Industrial Cryoconcentration of Grape (*Vitis Vinifera*) Must. J. Sci. Food Agric..

[17] Figueroa J.G., Borrás-Linares I., Lozano-Sánchez J., Segura-Carretero A. (2018). Comprehensive Characterization of Phenolic and Other Polar Compounds in the Seed and Seed Coat of Avocado by HPLC-DAD-ESI-QTOF-MS. Food Res. Int..

[18] Rodríguez-Carpena J.G., Morcuende D., Andrade M.J., Kylli P., Estevez M. (2011). Avocado (*Persea Americana* Mill.) Phenolics, in Vitro Antioxidant and Antimicrobial Activities, and Inhibition of Lipid and Protein Oxidation in Porcine Patties. J. Agric. Food Chem..

[19] Araújo R.G., Rodríguez-Jasso R.M., Ruiz H.A., Govea-Salas M., Pintado M., Aguilar C.N. (2021). Recovery of Bioactive Components from Avocado Peels Using Microwave-Assisted Extraction. Food Bioprod. Process..

[20] Samtiya M., Aluko R.E., Dhewa T., Moreno-Rojas J.M. (2021). Potential Health Benefits of Plant Food-Derived Bioactive Components: An Overview. Foods.

[21] Martínez-Patiño J.C., Gullón B., Romero I., Ruiz E., Brnčić M., Žlabur J.Š., Castro E. (2019). Optimization of Ultrasound-Assisted Extraction of Biomass from Olive Trees Using Response Surface Methodology. Ultrason. Sonochem..

[22] González-Centeno M.R., Knoerzer K., Sabarez H., Simal S., Rosselló C., Femenia A. (2014). Effect of Acoustic Frequency and Power Density on the Aqueous Ultrasonic-Assisted Extraction of Grape Pomace (*Vitis Vinifera* L.)—A Response Surface Approach. Ultrason. Sonochem..

[23] More P.R., Arya S.S. (2021). Intensification of Bio-Actives Extraction from Pomegranate Peel Using Pulsed Ultrasound: Effect of Factors, Correlation, Optimization and Antioxidant Bioactivities. Ultrason. Sonochem..

[24] Kumar K., Srivastav S., Sharanagat V.S. (2021). Ultrasound Assisted Extraction (UAE) of Bioactive Compounds from Fruit and Vegetable Processing by-Products: A Review. Ultrason. Sonochem..

[25] Chemat F., Rombaut N., Sicaire A.G., Meullemiestre A., Fabiano-Tixier A.S., Abert-Vian M. (2017). Ultrasound Assisted Extraction of Food and Natural Products. Mechanisms, Techniques, Combinations, Protocols and Applications. A Review. Ultrason. Sonochem..

[26] Roselló-Soto E., Galanakis C.M., Brnčić M., Orlien V., Trujillo F.J., Mawson R., Knoerzer K., Tiwari B.K., Barba F.J. (2015). Clean Recovery of Antioxidant Compounds from Plant Foods, by-Products and Algae Assisted by Ultrasounds Processing. Modeling Approaches to Optimize Processing Conditions. Trends Food Sci. Technol..

[27] Albahari P., Jug M., Radić K., Jurmanović S., Brnčić M., Brnčić S.R., Vitali Čepo D. (2018). Characterization of Olive Pomace Extract Obtained by Cyclodextrin-Enhanced Pulsed Ultrasound Assisted Extraction. LWT—Food Sci. Technol..

[28] Marić M., Grassino A.N., Zhu Z., Barba F.J., Brnčić M., Rimac Brnčić S. (2018). An Overview of the Traditional and Innovative Approaches for Pectin Extraction from Plant Food Wastes and By-Products: Ultrasound-, Microwaves-, and Enzyme-Assisted Extraction. Trends Food Sci. Technol..

[29] Tomšik A., Pavlić B., Vladić J., Ramić M., Brindza J., Vidović S. (2016). Optimization of Ultrasound-Assisted Extraction of Bioactive Compounds from Wild Garlic (*Allium Ursinum* L.). Ultrason. Sonochem..

[30] Gullón P., Gullón B., Astray G., Munekata P.E.S., Pateiro M., Lorenzo J.M. (2020). Value-Added Compound Recovery from Invasive Forest for Biofunctional Applications: Eucalyptus Species as a Case Study. Molecules.

[31] Chemat F., Rombaut N., Meullemiestre A., Turk M., Perino S., Fabiano-Tixier A.S., Abert-Vian M. (2017). Review of Green Food Processing Techniques. Preservation, Transformation, and Extraction. Innov. Food Sci. Emerg. Technol..

[32] Gullón B., Gullón P., Lú-Chau T.A., Moreira M.T., Lema J.M., Eibes G. (2017). Optimization of Solvent Extraction of Antioxidants from *Eucalyptus Globulus* Leaves by Response Surface Methodology: Characterization and Assessment of Their Bioactive Properties. Ind. Crop. Prod..

[33] Gullón B., Eibes G., Moreira M.T., Herrera R., Labidi J., Gullón P. (2018). Yerba Mate Waste: A Sustainable Resource of Antioxidant Compounds. Ind. Crop. Prod..

[34] Blasa M., Candiracci M., Accorsi A., Piacentini M.P., Albertini M.C., Piatti E. (2006). Raw Millefiori Honey Is Packed Full of Antioxidants. Food Chem..

[35] Ferreira-Santos P., Genisheva Z., Botelho C., Santos J., Ramos C., Teixeira J.A., Rocha C.M.R. (2020). Unravelling the Biological Potential of *Pinus Pinaster* Bark Extracts. Antioxidants.

[36] Helm K., Beyreis M., Mayr C., Ritter M., Jakab M., Kiesslich T., Plaetzer K. (2017). In Vitro Cell Death Discrimination and Screening Method by Simple and Cost-Effective Viability Analysis. Cell. Physiol. Biochem..

[37] Xu D.P., Zheng J., Zhou Y., Li Y., Li S., Li H. (2017). Bin. Ultrasound-Assisted Extraction of Natural Antioxidants from the Flower of Limonium Sinuatum: Optimization and Comparison with Conventional Methods. Food Chem..

[38] Mateos R., Baeza G., Sarriá B., Bravo L. (2018). Improved LC-MSn Characterization of Hydroxycinnamic Acid Derivatives and Flavonols in Different Commercial Mate (*Ilex Paraguariensis*) Brands. Quantification of Polyphenols, Methylxanthines, and Antioxidant Activity. Food Chem..

[39] Ballesteros L.F., Teixeira J.A., Mussatto S.I. (2014). Selection of the Solvent and Extraction Conditions for Maximum Recovery of Antioxidant Phenolic Compounds from Coffee Silverskin. Food Bioprocess Technol..

[40] Mokrani A., Madani K. (2016). Effect of Solvent, Time and Temperature on the Extraction of Phenolic Compounds and Antioxidant Capacity of Peach (*Prunus Persica* L.) Fruit. Sep. Purif. Technol..

[41] García-Castelló E.M., Rodríguez-López A.D., Mayor L., Ballesteros R., Conidi C., Cassano A. (2015). Optimization of Conventional and Ultrasound Assisted Extraction of Flavonoids from Grapefruit (*Citrus Paradisi* L.) Solid Wastes. LWT—Food Sci. Technol..

[42] Rico X., Gullón B., Yáñez R. (2020). Environmentally Friendly Hydrothermal Processing of Melon By-Products for the Recovery of Bioactive Pectic-Oligosaccharides. Foods.

[43] Segovia F.J., Corral-Pérez J.J., Almajano M.P. (2016). Avocado Seed: Modeling Extraction of Bioactive Compounds. Ind. Crop. Prod..

[44] Ilaiyaraja N., Likhith K.R., Sharath Babu G.R., Khanum F. (2015). Optimisation of Extraction of Bioactive Compounds from *Feronia Limonia* (Wood Apple) Fruit Using Response Surface Methodology (RSM). Food Chem..

[45] Dahmoune F., Boulekbache L., Moussi K., Aoun O., Spigno G., Madani K. (2013). Valorization of Citrus Limon Residues for the Recovery of Antioxidants: Evaluation and Optimization of Microwave and Ultrasound Application to Solvent Extraction. Ind. Crop. Prod..

[46] Araújo R.G., Rodríguez-Jasso R.M., Ruiz H.A., Govea-Salas M., Pintado M.E., Aguilar C.N. (2020). Process Optimization of Microwave-Assisted Extraction of Bioactive Molecules from Avocado Seeds. Ind. Crop. Prod..

[47] Rosero J.C., Cruz S., Osorio C., Hurtado N. (2019). Analysis of Phenolic Composition of Byproducts (Seeds and Peels) of Avocado (*Persea Americana* Mill.) Cultivated in Colombia. Molecules.

[48] Kosińska A., Karamać M., Estrella I., Hernández T., Bartolomé B., Dykes G.A. (2012). Phenolic Compound Profiles and Antioxidant Capacity of *Persea Americana* Mill. Peels and Seeds of Two Varieties. J. Agric. Food Chem..

[49] López-Cobo A., Gómez-Caravaca A.M., Pasini F., Caboni M.F., Segura-Carretero A., Fernández-Gutiérrez A. (2016). HPLC-DAD-ESI-QTOF-MS and HPLC-FLD-MS as Valuable Tools for the Determination of Phenolic and Other Polar Compounds in the Edible Part and by-Products of Avocado. LWT—Food Sci. Technol..

[50] Ibrahima R.M., El-Halawany A.M., Saleh D.O., El Naggar E.M.B., EL-Shabrawy A.E.R.O., El-Hawary S.S. (2015). HPLC-DAD-MS/MS Profiling of Phenolics from *Securigera Securidaca* Flowers and Its Anti-Hyperglycemic and Anti-Hyperlipidemic Activities. Rev. Bras. Farmacogn..

[51] De Araújo F.F., De Paulo Farias D., Neri-Numa I.A., Pastore G.M. (2021). Polyphenols and Their Applications: An Approach in Food Chemistry and Innovation Potential. Food Chem..

[52] Puiggròs F., Llópiz N., Ardévol A., Bladé C., Arola L., Salvadó M.J. (2005). Grape Seed Procyanidins Prevent Oxidative Injury by Modulating the Expression of Antioxidant Enzyme Systems. J. Agric. Food Chem..

[53] Jeong W.-S., Kong A.-N.T. (2004). Biological Properties of Monomeric and Polymeric Catechins: Green Tea Catechins and Procyanidins. Pharm. Biol..

[54] Howell A.B., Reed J.D., Krueger C.G., Winterbottom R., Cunningham D.G., Leahy M. (2005). A-Type Cranberry Proanthocyanidins and Uropathogenic Bacterial Anti-Adhesion Activity. Phytochemistry.

[55] Kalisz G., Gieroba B., Chrobak O., Suchora M., Starosta A.L., Sroka-Bartnicka A. (2021). Vibrational Spectroscopic Analyses and Imaging of the Early Middle Ages Hemp Bast Fibres Recovered from Lake Sediments. Molecules.

[56] Firdaus M.L., Fitriani I., Wyantuti S., Hartati Y.W., Khaydarov R., Mcalister J.A., Obata H., Gamo T. (2017). Colorimetric Detection of Mercury(II) Ion in Aqueous Solution Using Silver Nanoparticles. Anal. Sci..

[57] Lombardi V.R.M., Carrera I., Cacabelos R. (2017). In Vitro Screening for Cytotoxic Activity of Herbal Extracts. Evid.-Based Complement. Altern. Med..

[58] Bhuyan D.J., Alsherbiny M.A., Perera S., Low M., Basu A., Devi O.A., Barooah M.S., Li C.G., Papoutsis K. (2019). The Odyssey of Bioactive Compounds in Avocado (*Persea Americana*) and Their Health Benefits. Antioxidants.

[59] Bhosale P.B., Ha S.E., Vetrivel P., Kim H.H., Kim S.M., Kim G.S. (2020). Functions of Polyphenols and Its Anticancer Properties in Biomedical Research: A Narrative Review. Transl. Cancer Res..

[60] Niedzwiecki A., Roomi M.W., Kalinovsky T., Rath M. (2016). Anticancer Efficacy of Polyphenols and Their Combinations. Nutrients.

[61] Nabavi S.F., Nabavi S.M., Setzer W.N., Nabavi S.A., Nabavi S.A., Ebrahimzadeh M.A. (2013). Antioxidant and Antihemolytic Activity of Lipid-Soluble Bioactive Substances in Avocado Fruits. Fruits.

[62] Brooke D.G., Shelley E.J., Roberts C.G., Denny W.A., Sutherland R.L., Butt A.J. (2011). Synthesis and in Vitro Evaluation of Analogues of Avocado-Produced Toxin (+)-(R)-Persin in Human Breast Cancer Cells. Bioorg. Med. Chem..

[63] León L.G., Carballo R.M., Vega-Hernández M.C., Miranda P.O., Martín V.S., Padrón J.I., Padrón J.M. (2008). Β′-Hydroxy-α,β-Unsaturated Ketones: A New Pharmacophore for the Design of Anticancer Drugs. Part 2. ChemMedChem.

[64] Guzmán-Rodríguez J.J., López-Gómez R., Salgado-Garciglia R., Ochoa-Zarzosa A., López-Meza J.E. (2016). The Defensin from Avocado (Persea Americana Var. Drymifolia) PaDef Induces Apoptosis in the Human Breast Cancer Cell Line MCF-7. Biomed. Pharmacother..

[65] Flores-Álvarez L.J., Guzmán-Rodríguez J.J., López-Gómez R., Salgado-Garciglia R., Ochoa-Zarzosa A., López-Meza J.E. (2018). PaDef Defensin from Avocado (*Persea Americana* Var. Drymifolia) Is Cytotoxic to K562 Chronic Myeloid Leukemia Cells through Extrinsic Apoptosis. Int. J. Biochem. Cell Biol..

[66] Bhattacharyya S.S., Paul S., Dutta S., Boujedaini N., Khuda-Bukhsh A.R. (2010). Anti-Oncogenic Potentials of a Plant Coumarin (7-Hydroxy-6-Methoxy Coumarin) against 7,12-Dimethylbenz[a]Anthracene-Induced Skin Papilloma in Mice: The Possible Role of Several Key Signal Proteins. J. Chin. Integr. Med..

[67] Riss T.L., Moravec R.A., Niles A.L., Duellman S., Benink H.A., Worzella T.J., Minor L. (2016). Cell Viability Assays. Assay Guidance Manual.

[68] Sun H., Yin M., Hao D., Shen Y. (2020). Anti-Cancer Activity of Catechin against A549 Lung Carcinoma Cells by Induction of Cyclin Kinase Inhibitor P21 and Suppression of Cyclin E1 and P-AKT. Appl. Sci..

[69] Imran M., Salehi B., Sharifi-Rad J., Gondal T.A., Saeed F., Imran A., Shahbaz M., Fokou P.V.T., Arshad M.U., Khan H. (2019). Kaempferol: A Key Emphasis to Its Anticancer Potential. Molecules.

[70] Rauf A., Imran M., Khan I.A., ur-Rehman M., Gilani S.A., Mehmood Z., Mubarak M.S. (2018). Anticancer Potential of Quercetin: A Comprehensive Review. Phyther. Res..

